# HiTSEE KNIME: a visualization tool for hit selection and analysis in high-throughput screening experiments for the KNIME platform

**DOI:** 10.1186/1471-2105-13-S8-S4

**Published:** 2012-05-18

**Authors:** Hendrik Strobelt, Enrico Bertini, Joachim Braun, Oliver Deussen, Ulrich Groth, Thomas U Mayer, Dorit Merhof

**Affiliations:** 1 Department of Computer Science, Konstanz University, Universitätsstr. 10, Konstanz, Germany; 2 Konstanz Research School Chemical Biology, Konstanz University, Universitätsstr. 10, Konstanz, Germany; 3 Interdisciplinary Center for Interactive Data Analysis, Modelling and Visual Exploration (INCIDE), Konstanz University, Universitätsstr. 10, Konstanz, Germany

## Abstract

We present HiTSEE (High-Throughput Screening Exploration Environment), a visualization tool for the analysis of large chemical screens used to examine biochemical processes. The tool supports the investigation of structure-activity relationships (SAR analysis) and, through a flexible interaction mechanism, the navigation of large chemical spaces. Our approach is based on the projection of one or a few molecules of interest and the expansion around their neighborhood and allows for the exploration of large chemical libraries without the need to create an all encompassing overview of the whole library. We describe the requirements we collected during our collaboration with biologists and chemists, the design rationale behind the tool, and two case studies on different datasets. The described integration (HiTSEE KNIME) into the KNIME platform allows additional flexibility in adopting our approach to a wide range of different biochemical problems and enables other research groups to use HiTSEE.

## Introduction

Genetics has been widely used in the past to study complex biological processes within a cellular system and to elucidate the function of proteins. As genes encode proteins, gene function can be modulated through a mutation, which in turn perturbs the function of the protein of interest and either affects its activity or entirely suppresses its expression ("knockout"). As a result, the physiological effect observed in the phenotype allows the protein function to be identified.

Although genetic approaches have proven to be extremely powerful in elucidating the principles of a wide range of biological processes, there are a number of substantial limitations to this approach, most importantly the lack of temporal control required to study dynamic processes, since a protein cannot be turned on or off on demand. A more recent approach to study protein function, which overcomes this limitation, is chemical genetics. In chemical genetics, biological systems are studied using cell-permeable small molecules (compounds), which inhibit the protein under investigation (chemical knock-out). This approach makes it possible to perturb protein function rapidly, reversibly and conditionally with temporal and quantitative control, both in cultured cells or whole organisms [1].

The foundation of chemical screens are commercially available compound libraries comprising hundreds of thousands of small molecules that cover a high degree of structural diversity. In order to switch a protein off, a compound needs to be identified that inhibits the protein under investigation and hence allows its function to be studied. For this purpose, high-throughput screening (HTS) is performed. This is a major technological breakthrough in biology experimentation [2].

Although experimentation capabilities have increased significantly over the last years, resulting in vast amounts of data generated in high-throughput screenings, the development of analysis methods that are able to handle and process large amounts of data is lagging behind and does not scale at any equally fast rate. For this reason, many sites that deploy high-throughput screenings use sub-optimal solutions which are either too slow or suffer from a limited scope of analysis.

The development of HiTSEE stems from the analysis of HTS data analysis practices performed by several researchers at the School of Chemical Biology at the University of Konstanz and from the analysis of existing HTS tools. We discovered that electronic spreadsheets are the main data analysis tool employed by the researchers and that their data exploration capabilities are, as a consequence, extremely limited. These practices not only leave room to several kinds of mistakes, but they also hinder the possibility of effectively exploring the chemical space and relating activity levels to structural features.

At the same time, all the tools we have analyzed did not completely fit the needs of our researchers. While the whole field of Chemoinformatics has developed numerous and impressive computational tools for drug discovery (mainly in the pharmaceutical industry), there is a lack of flexible visualization tools that allow for the lower-scale smooth exploration of chemical spaces. During our analysis we reviewed a number of visualization tools for structure-activity relationships (we provide a full description and comparison in the Related Work Section) but none of them seemed to fit the needs we encountered. We believe this is due to three main factors: (1) the tools tend to focus either on gaining an overview of a chemical space or on the exploration of the neighborhood of a single compound; (2) the tools tend to focus either on the comparison of entire molecules or on their fragments; (3) many tools offer limited navigation and interaction capabilities.

HiTSEE addresses these issues by providing a multi-view interactive system in which it is possible to project one or more compounds of interest and explore a neighborhood. The tool features flexible navigation capabilities that allow the user to easily jump from one chemical context to another.

The main contributions of this paper are: the in-depth analysis of the HTS problem with a group of researchers involved in biochemistry, the design rationale and development of a flexible visual HTS analysis tool, and its interaction paradigm within KNIME [3].

The validity of HiTSEE (KNIME) is demonstrated by two case studies performed by biochemistry experts. The presented approach is of major interest for biologists involved in high-throughput experiments and visualization designers that want to learn from a real design study.

The paper is organized as follows: the following section describes HTS in more details to provide the right context to readers not familiar with the process, the *Data Processing and Tasks *Subsection describes the data processing steps needed before the data could enter into the system and the tasks collected during our collaboration, the *HiTSEE *Section describes HiTSEE and its design, the *HiTSEE for KNIME *Section gives detailed information on the implementation and use of HiTSEE within the KNIME platform, the *Results *Section illustrates the case studies, the *Conclusion *Section offers some reflections and lessons learn from the process and outlines our plans for future work.

### Related work

While there are a number of free and commercial tools that support one or more phases of HTS (e.g., Spotfire), in the following we focus our review on visualization tools that specifically address hit selection, exploration and expansion and more specifically the understanding of structure-activity relationships.

SARANEA [4] is a visualization tool to support structure-activity relationship and selectivity analysis. It is based on a network graph visualization. The graph is built by connecting molecules with an edge if their similarity value is higher than a predefined threshold and projected using the classical Fruchterman-Reingold algorithm. The main feature of the tool is the calculation and visualization (through color) of a "cliff index", which describes whether the compound has a big shift in potency compared to its neighborhood. HiTSEE can also help in the detection of activity cliffs by spotting big changes in size within a given cluster. While SARANEA is more targeted towards the exploration of a full set of compounds, HiTSEE leverages on the idea of having a small set of initial compounds (often a single one) and to explore their direct neighborhood.

Scaffold Hunter [5] is another visualization tool that can be used to find relations between structural features and activity level. A tree structure is built starting from one compound and building molecular scaffolds by removing rings in the periphery, through a series of chemistry and medicinal chemistry rules. The visual representation is a radial tree depicting the hierarchical scaffold structure and the activity level of the scaffolds. The tool can be used to investigate the potential of the scaffold to be at the origin of activation of biological processes. Together with HiTSEE, Scaffold Hunter shares the idea of starting the analysis from one (or more in HiTSEE) compounds of interest and exploring their neighborhood. However, HiTSEE is focused more on structural similarities between the compounds rather than the scaffolds.

DrugViz [6] is a newly developed plug-in in the Cytoscape environment in which analysis is centered on a network representation of interactions between biological targets. The system enables similar targets to be picked and common compounds to be found, or alternatively, the system selects similar compounds and sees how they are distributed in the target network. While the system allows the investigation of relationships between biological targets and molecular structures, visualization is not targeted towards the visualization of structure-activity relationships.

The SAR Map [7] (and its extension Enhanced SAR Map) allows the focus to lie on one single molecule of interest, exploring all its substituents through R-group analysis. For its input, R-group analysis takes a list of compounds with a common scaffold and generates all possible variations. The SAR map is a heat map where the columns and the rows represent substituents of two selected variation sites. Each cell represents one specific compound (formed by attaching the substituents) and a color map, or more complex visualizations provide rich information about each compound. HiTSEE also allows the variations around a subset of structurally similar compounds (in the molecule details view) to be visualized, however this functionality takes place in the larger context of similarity analysis among entire molecules.

ChemGPS-NP [8], similarly to HiTSEE, projects molecules in a low-dimensional space using a PCA projection. The visualization is designed in a way so as to reflect those properties that are relevant for bioactivity. However, the visualization does not directly adress the correlation between activity and structural similarity.

In summary, HiTSEE has the unique advantage of allowing flexible and smooth navigation in the chemical space by conjugating two contrasting needs: the need to create visual summaries of chemical libraries and the need to explore the neighborhood of selected compounds.

## Methods and data

HTS provides a means to quickly test a large number of chemical compounds against a biological target in order to determine potentially interesting compounds (hits) which can then be investigated further. Once the data has been collected, the researcher must go through the following stages:

### 

#### Data processing & quality control

The researcher normalizes the data against the control values and analyzes the result to check for abnormal behaviors. A number of biases and outliers can exist in an HTS experiment. In our environment we typically check for assay plates with a low Z-factor [9], a measure of assay quality based on the controls. This phase is also supported by a visual tool we have developed previously [10] to visually explore the values in the plates. The researchers can directly filter out those compounds with unreliable readouts or simply mark them for future analysis.

#### Hit selection

The goal of this phase is to identify the compounds that reacted in the experiment. Typically, the researcher organizes the results in a list sorted according to activity level and chooses a threshold value above which the compounds are considered active. These compounds are called *hits*.

#### Hit confirmation

After hit identification, the researcher re-tests the selected hits in a new and more focused screening (e.g. testing different concentrations of the compounds and calculating the *half maximal inhibitory concentration *(*IC*_50_)) in which confirmation of activity is sought. This phase is normally done manually given the small number of molecules to test.

#### Hit exploration

After hit confirmation the researcher explores the chemical space around the hits. Typically, he or she is in search of relationships between molecular structure and activity to isolate molecular fragments that induce activity (a process called *structure-activity relationship (SAR) analysis*).

#### Hit expansion

Similar to hit exploration, hit expansion focuses on exploring the space around the hits but the focus is on identifying alternative molecules that retain the desired properties and meet additional requirements (e.g. solubility).

Each one of these steps can involve data and computational resources. While in our environment we provide support for all these stages, HiTSEE provides only support for a subset of them, namely: *selection*, *exploration*, and *expansion*.

### Data processing and tasks

In the following we provide additional details about the data and describe how it is processed before entering into the description of HiTSEE. Then, we describe how we gathered the requirements for the design of the tool and discuss our main motivation for focusing on a subset of the HTS tasks.

#### Data processing

HiTSEE KNIME integrates fully into the KNIME platform [3]. KNIME is a well-known data mining framework based on a workflow paradigm where data is processed by connecting data processing *nodes *one to another. It features an extensive and extensible library of nodes with a variety of purposes, e.g., querying, data mining algorithms, biochemical libraries. Before entering the HiTSEE KNIME nodes (more details in Section *HiTSEE for KNIME*) additional nodes preprocess the data within the platform:

1. *Data Normalization *- The system enables several kinds of normalization to be applied and take different plate formats into account. Typically at this stage the system normalizes the data, taking the values found in the control cells into consideration, and using the average value of the positive and negative controls.

2. *Quality Control *- At this stage the researcher can use a variety of tools we have developed to asses the quality of the experiment and to filter out or mark values with unusual behaviors. Many of the functions we have implemented leverage on a plate view through which the user can observe the distribution of the activity levels across the plates.

3. *Fingerprint Generation *- The molecular structure of each compound (described by the SMILE format in the database) has to be translated into a format that allows structural similarity comparisons between the molecules. Thus, we transform the molecular descriptions into binary vectors called *fingerprints*.

Since this last fingerprint generation step is critical for the way HiTSEE arranges the molecules in its main view we provide additional details about it below.

#### Fingerprint generation

Chemoinformatics applications use fingerprints (FPs) as a way to allow similarity searches and comparisons between molecules. The basic idea behind FPs is to describe each molecule with a numeric vector that captures relevant properties of molecules. While FPs can capture a variety of molecular features, structural fingerprints are above all the most popular [11]. Structural fingerprints are based on the concept of molecular fragments, that is, subsets of atoms and bonds found in the original sets, and describe each molecule in terms of the presence or absence of a molecular fragment. A fingerprint is thus a (normally very long) binary vector where each entry represents a fragment. The value is set to one if the fragment is contained in the molecule and to zero otherwise. Through such a binary representation it is possible to compare the structural similarity of molecules: two molecules with similar vectors contain similar molecular fragments.

For more information on fingerprints and related techniques in chemoinformatics Leach and Gillet [12] provide an excellent introduction to the aforementioned concepts. Bender and Glen [13] provide an overview on molecule similarity measures (with and without the use of fingerprints) which can partially be applied using HiTSEE in the KNIME environment. Additionally, thorough reviews on fingerprints and chemical similarity can be found in the following papers: [14-16].

#### Tasks

The requirements we have gathered to design HiTSEE are the result of a long-term collaboration between the Department of Information Science and the Konstanz Research School in Chemical Biology at the University of Konstanz. We organized regular meetings between the involved groups to become acquainted with the biochemical problems and to gather information about current practices and data analysis needs. HiTSEE is the last in a number of developed prototypes designed over a year and a half of collaboration. We used the prototypes as a way to probe the design space, to better understand the domain problems, and eventually to isolate the tasks that needed a real support in terms of visual analytics tools.

While we originally developed prototypes for a diverse number of tasks throughout the range of the HTS steps, e.g., data processing, quality control, and chemical libraries overviews, HiTSEE has been designed specifically to support hit selection, exploration and expansion. More precisely we provide support for two main visual analytics tasks:

1. *Setting a threshold in hit selection*. One of the challenges we encountered early on in the process was the definition of an activity threshold value in the hit selection process. From our observations and discussions with the domain experts we realized that the hits are normally selected through a fuzzy process. The researcher sorts the molecules according to their activity value and chooses a threshold going by eye, searching for a trade-off between the number of hits (to be kept low for later, more in-depth, testing) and the risk of missing important molecules. One need voiced by our collaborators was the possibility to gain, already at this stage, a better view on the selected hits in order to make the hit selection process more informed.

2. *Exploring the neighborhood of confirmed hits*. A second major need we spotted during our collaboration consists of the exploration of the neighborhood of one or more confirmed hits in the hit expansion phase. This stage starts when one or more molecules are declared to be active in a secondary screening. At this point, the researcher wants to explore the neighborhood to: (1) understand how little structural changes influence the chemical behavior with the selected target; (2) find a trade-off between the activity level expressed by the compounds and other chemical features of interest. In our specific case, for instance, the solubility of the compounds (measured in LogP values) is a critical element to isolate molecules of interest.

HiTSEE supports these two tasks in an integrated environment in which the user can project elements of interest in a scatter plot view, expand the projected items to include their neighbors, and perform several interactive operations that support flexible navigation and details on demand. In the following we describe HiTSEE in detail and explain how it supports the aforementioned tasks.

### HiTSEE

**Figure 1:**
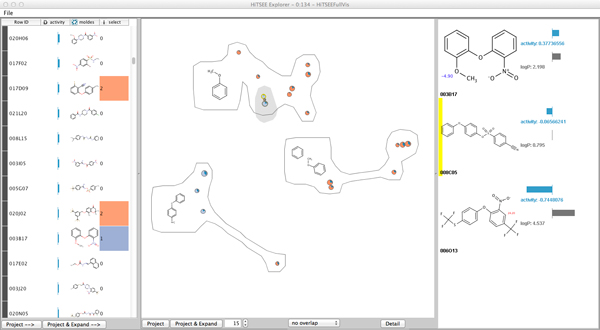
**HiTSEE main view**. The Main View of HiTSEE KNIME containing the *list view *(left), the *projection view *(middle), and the *detail view *(right). The gray selected molecules are input to the *detail view*. The hovered item in the *detail view *is highlighted yellow in the *projection view*.

**Figure 2:**
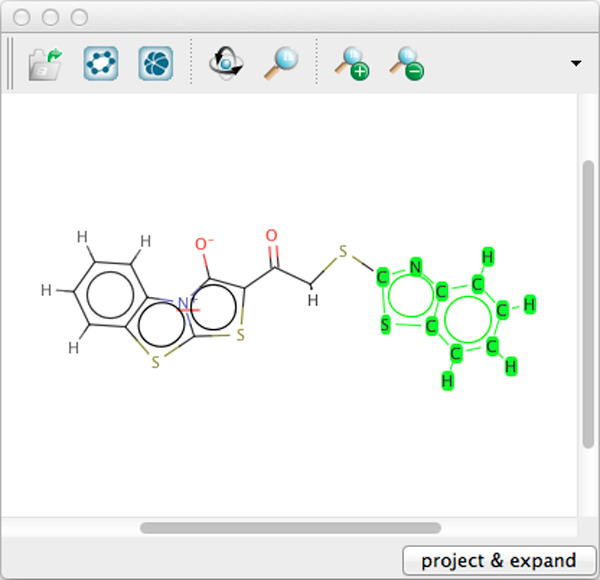
**HiTSEE substructure search view**. The HiTSEE *HiTSEE substructure search view *derived from JChem Marvin Sketch Applet.

HiTSEE's interface is organized around three main views: list+projection view (Figure 1 (left, middle)), molecules detail (Figure 1 (right)) and substructure search view (Figure 2), that support exploration, in-depth investigation and structural queries. The *list+projection view *permits molecules of interest to be selected and to project them in a scatter plot visualization to form clusters of (structurally) similar compounds. The view supports the investigation of relationships between activity levels, structural features, and other chemical properties. The *molecules detail and substructure search view *shows the molecular structure of compounds selected in the projection view and triggers substructure searches.

In the following we describe each component in detail together with the interaction capabilities offered by each one.

#### List+Projection view

**Figure 3:**
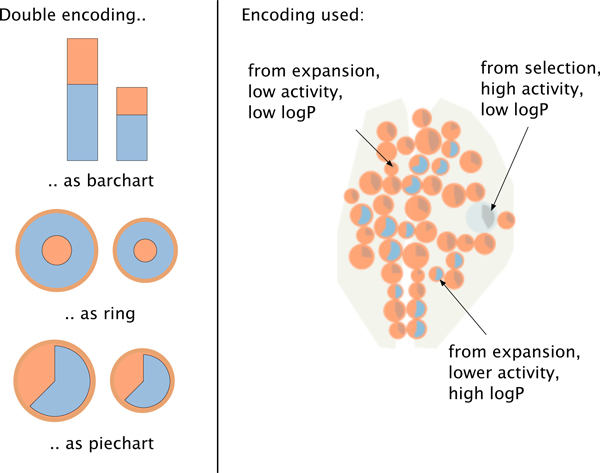
**Visual encoding in HiTSEE**. *Left: *Different alternatives for visually encoding activity (length/radius) and logP (proportion of shape). *Right: *HiTSEE's mapping of origin (direct selection or expansion), activity level, and logP value to the visual features: color (orange, blue), size (circle radius), and angle (pie chart).

The List+Projection view consists of two interactive elements: a compounds list and a linked scatter plot view. (Figure 3 (left)) The compound list organizes the full set of compounds in the library in a list format sorted by activity level. Each item is represented by its molecular structure and by a bar with length proportional to its activity level.

The user can select one or more items in the list, project them in the scatter plot view, and expand the selection to a user-defined number of neighbors. The neighbors are the compounds that are structurally most similar to the current selection. The structural similarity is calculated from the fingerprint bit-vectors (see Fingerprint Section).

The compounds are represented by circles and positioned in the view through a multidimensional scaling (MDS) projection such that compounds with similar structures occupy similar positions. Size represents the activity level and color is used to distinguish between those compounds included in the initial selection and those added by the expansion mechanism. Each circle also contains a small modified pie chart representing additional chemical properties of interest (in our case the LogP value). The pie chart is designed in a way to turn its fill color into a more prominent one (darker blue) when the value of interest goes beyond a predefined threshold.

The MDS projection takes a distance matrix of metric distance values as input. For each pair of compounds, we calculate the Tanimoto [17] distance between their fingerprint bit-vectors. Two problems emerge from MDS-based projections: overlapping items and fuzzy boundaries between the groupings. To cope with these two issues we implemented two additional features. First, we used an overlap removal mechanism that permits to displace the items from their original position if they overlap each other. Second, in order to facilitate the grouping of the items, we cluster the items and draw a "bubble" around them to reinforce the perception of grouping. The clustering algorithm takes the screen-space positions of the items as input and clusters them into bubble sets [18]. For each cluster, we determine the common substructure of all containing compounds and position it left to the cluster.

In designing the projection view, we tried to optimize its visual effectiveness towards reading patterns with biological interest. In the following we provide a summary of the rationale behind our main design choices.

Since position is the visual variable that can be perceived pre-attentively most accurate [19], we use it to convey molecular similarity (through the proximity data given by MDS), which is the most important piece of information in the data. Activity level is mapped to circle size (with a square root mapping to take into account the area effect) to allow for easy discrimination among the molecules. While visual variables like bar length allow for a more accurate comparison of values [19], we decided to use circles and their size because: (1) they cluster more naturally than shapes with other aspect ratios, (2) they are more robust w.r.t. the overlapping removal mechanism, (3) they allow for easy discrimination between high vs. low activity molecules while keeping the visualization compact, (4) reading the activity values accurately is not the main purpose of the visualization (as long as major differences can be spotted). A third parameter (LogP) is encoded as the visual variable *angle*. To allow better readability we visualize the angle by using a filled pie chart with only one pie embedded in the circles. While a number of alternatives exist to encode two parameters, as for instance stacked bars and nested circles (see Figure 3), we decided to use a modified pie chart because it corresponds well with the circular shape we adopted and readability scales visually better than nested rings to items of different size.

#### Molecules detail view and substructure search view

From the projection view the user can select a group of interesting compounds to be investigated in detail. Figure 1 (right) shows the detail view with its core features.

The selected set of compounds is visualized as an ordered list of high resolution molecule renderings. We map the chemical features *activity *and *logP *into small bar charts to the right, the *pKa *values are rendered directly into the molecule.

During the investigation of the molecules we permit the user to start a search on a particular pattern by selecting a molecular fragment and issuing a query for retrieving all the compounds containing the selected fragment. We support this function by providing the substructure search view, which opens when the user double-clicks on a molecule in the detail view.

The substructure search view (Figure 2) is based on the JChem Marvin Sketch applet (see Section *Implementation Details*), which provides a common interactive method for selecting substructures. The user starts a search on the selected substructure, the search results are highlighted as selections in the List+Projection view, and the user can project them in the projection view for investigation.

### HiTSEE for KNIME

**Figure 4:**
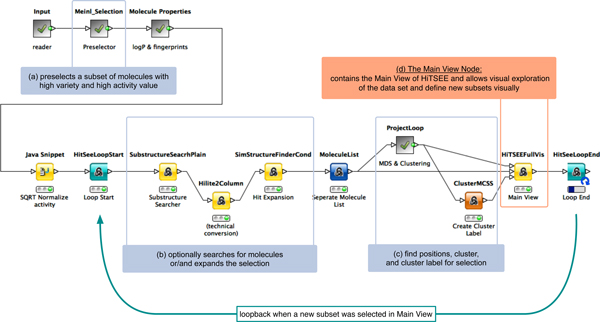
**HiTSEE as KNIME workflow**. An example of a KNIME workflow using the HiTSEE extensions. The data input and data preparation nodes are shown in the first row. Nodes for the iterative loop to select subsets of the given molecule library visually, are shown in the lower part.

Integration of HiTSEE into the KNIME platform is achieved by the development of a series of processing nodes that realize the functions developed in HiTSEE and additional helper nodes that permit to build a fully functional pipeline. Figure 4 shows an example of the workflow. The data can be read and pre-processed using the large set of available nodes in the KNIME platform (many specifically built for processing biological data). In the example, the first set of nodes permits to load the data into the system; preprocess it to calculate derived information, such as logP and fingerprints; and to apply some normalization functions. The nodes between the "Loop Start" and "Loop End" represent the core functionalities offered by the HiTSEE KNIME integration and reflect the main behavior of HiTSEE as a continuous iteration loop to focus on a specific set of compounds.

Each iteration starts with the selection of a molecule subset from the user directly or indirect from a user defined search request. The first set of nodes (b) is responsible for the generation of the expanded set (for a project-and-expand operation) and for the search of the target molecules of a substructure search. The second set of nodes (c) is responsible for projection and clustering. More specifically, this node implements the following set of functions: (1) calculate the distances between the molecules in the selected (and expanded) subset; (2) project the distances; (3) cluster the projected positions; (4) find a most common substructure as cluster label. Finally, the last node (d) is the one that implements the visualization, the interactive functions, and the loop control.

The "Project" and "Project and Expand" buttons trigger the HiTSEE loop and permit it to execute one iteration of the steps described above under the parameters given by the user. The main view is split into the previously described *list view*, *projection view*, and *detail view*. The example in Figure 1 shows a selection made by the user (highlighted in gray). When the "Project & Expand" button is clicked, the following parameters are set for the next loop cycle. The three selected molecules are the new subset of interest, an expansion of the subset is required for a set size of 15 additional compounds. The loop is started with these parameters for one cycle and the main view changes accordingly.

#### Flexibility

Integration into the KNIME platform allows a higher flexibility in changing the behavior of HiTSEE. The major advantage rests in the fact that the processing work flow can be easily changed. In the following, we provide a few examples of changes that could be applied by simply replacing or adding nodes in the workflow:

• The *distance measure *between molecules can be changed. The originally used Tanimoto metric to calculate the similarity between the molecules can be substituted by calculations of alternative fingerprints or alternative metrics.

• The *activity value *can be transformed, e.g., to reduce highly dynamic behaviors. This is exemplified in Figure 4 by applying a square root scaling to the activity value (see "SQRT normalize activity" node before the loop start).

• *Data preprocessing *can be modified and allows richer automation. We applied an algorithm from Meinl et.al. [20] to initially select molecules of high structural diversity with a high activity value. (see "Meinl Selection" in Figure 4).

Many more functions can be integrated according to the specific needs of the analysis and the provision of nodes from the KNIME platform. By the integration and deployment of HiTSEE KNIME we reach a higher degree of generalization and enable our approach to be adopted for a wider range of biochemical challenges.

#### Implementation details

The KNIME version of HiTSEE is programmed in Java. For rendering molecules, finding common substructures, and making the interactive selection we use the KNIME integration of the JChem library version 5.4, 2011, ChemAxon http://www.chemaxon.com and infocom (http://infocom-science.jp/product/detail/jchemextensions_english.html). For the implementation of visual components we use the Processing http://processing.org/ framework v1.0 and the giCentreUtils v3.1.0 http://www.gicentre.org/utils/. MDS projection is performed by the Java Library for Multidimensional Scaling v0.2 http://www.inf.uni-konstanz.de/algo/software/mdsj/. The cluster shapes are generated with BubbleSets https://github.com/JosuaKrause/Bubble-Sets.

## Results

We conduct two case studies applying different screening datasets to our prototype and to the KNIME version of HiTSEE: We describe the case study for an ongoing research project in our Biochemistry group (Kif18A data set). To show general applicability we provide a case study for using HiTSEE KNIME for the NCI AIDS Antiviral Screen.

### Case study 1: the Kif18A data set

We used HTS-data generated by a screen looking for a specific inhibitor of Kif18A [21] to prove the effectiveness and usability of HiTSEE.

Kif18A belongs to the family of mitotic kinesins. Kinesins are ATP dependent motor proteins, which utilize the energy derived from ATP hydrolysis to produce mechanical force. Kif18A belongs to the kinesin-8 family whose members are known to be required for the correct segregation of chromosomes in mitosis. Besides its key function in mitosis, Kif18A is characterized by its unique enzymatic properties since it integrates both motility and microtubule depolymerization activity. Due to its central function in mitosis and intriguing enzymatic properties we applied a small molecule screen to identify small molecules that inhibit the ATPase activity of Kif18A. The published results of the small molecule screen [21] can be applied as a proof-of-concept principle to validate HiTSEE.

### Case study 1: hit selection

**Figure 5:**
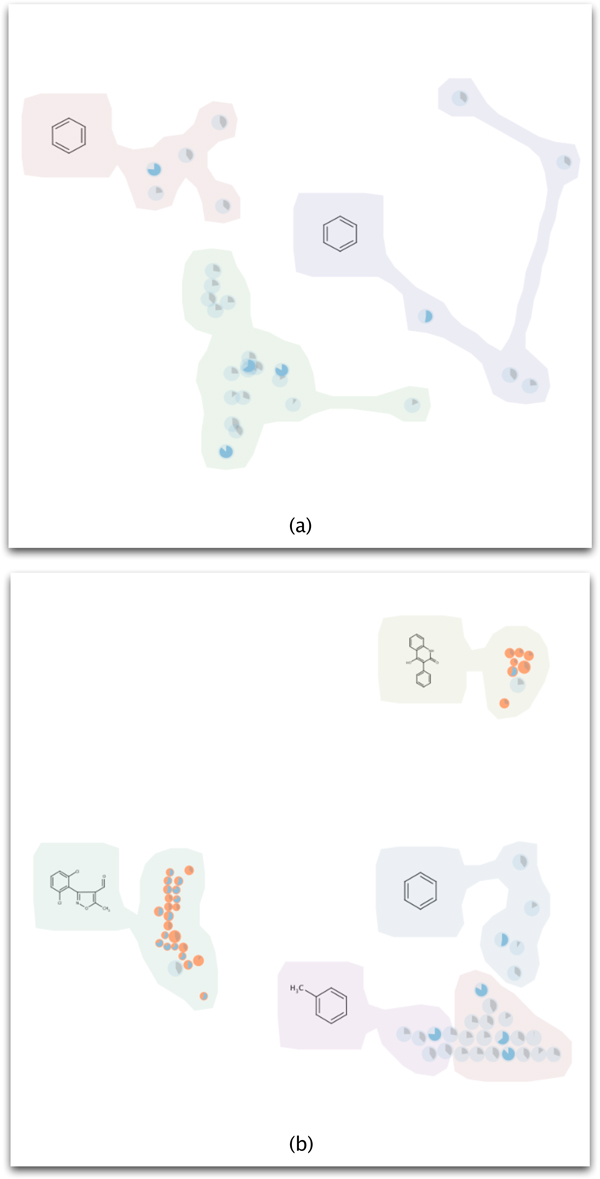
**Case study 1a**. The *projection view *for case study *Hit Selection*. In a) the projection of the users original selection is shown. The only common motif is the phenyl moiety. That this motif is shown in two clusters can be interpreted as: "The contained compounds share a common substructure, but besides that they form different clusters of structural similarity." By expanding the selection in b) a hit can be visually indicated, because the structural similar motifs are spread over an activity range.

The first step after a high throughput screen is to decide which positive results are counted as a hit, whereupon the compounds are sorted according to their activity level. With HiTSEE the compounds are directly sorted according to their activity level and the corresponding structures are represented in a list. After choosing the 30 compounds with the highest activity and projecting them, we have a first set for hit selection. In the projection we could see the common structure of the clusters (Figure 5a). The only common motif in this case was the phenyl moiety, which is not really significant. Also blue filling of the dots indicates a LogP value above 5, which could cause solubility problems in aqueous media. Nevertheless, by removing the overlap we are able to see the structure of the active compounds and get first hints for structures relevant for activity. In the detailed view of all these selected compounds we can easily compare their structures by eye and spot new common or interesting structures like the diphenyl sulfide moiety. With the project and expand option we were able to see structures related to the hits with lower activity levels (indicated by orange dots, Figure 5b). Clusters of highly active and less active compounds made us feel more confident to select the highly active compound as a hit, because the structural motif is spread over an activity range.

### Case study 1: hit expansion

**Figure 6:**
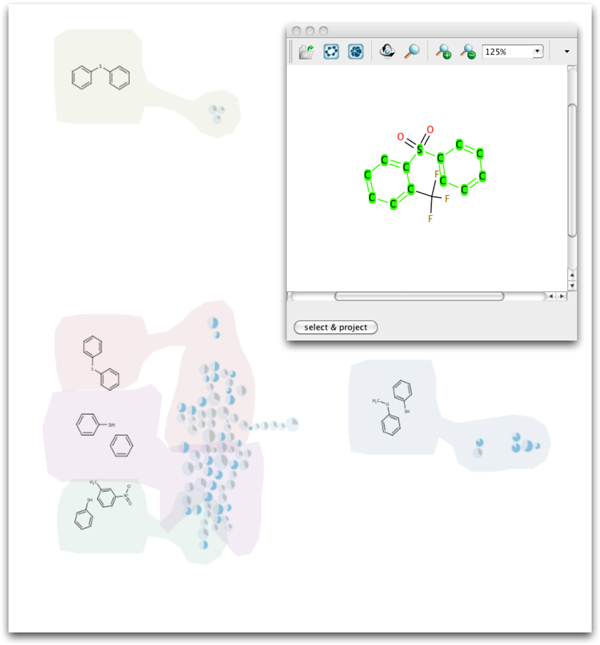
**Case study 1b**. The *projection view *and *substructure search view *(inset) for case study *Hit Expansion*.

After one hit has been selected we can choose structural elements and search the entire library for that motif. In our case we started by projecting the 30 compounds with the highest activity in the view. After selecting all the hits and going through the detailed view we decided to look for compounds containing a diphenyl sulfide moiety, which was present in several hits (Figure 6). The result was that we received nice clusters of compounds. This clustering gives us more confidence to choose the selected hit for further testing because now we know that there are other compounds containing the same structural moiety with a different activity level. This activity range allows us to establish SARs, identify the core structure necessary for activity and hint to structures with higher activity. Further this finding appears to indicate that the selected hit is not a false positive because the substructure is present in different compounds. Using the detailed view of a cluster, a list of compounds with common substructures and diverse activity levels is obtained. The fact that the common motif is not only present in the highly active compounds but also in less active ones enabled us to establish SARs first and to feel confident in choosing this compound for further investigations. The search did not give BTB-1 as a result but it gave a whole set of BTB-1 analogues, which are also very active. If we were to continue with these results we would choose the diphenyl sulfone moiety as the lead structure, the published Kif18A inhibitor [21]. After investigating compounds with this structural motif and establishing SARs we would finally end at BTB-1. HiTSEE allows hits to be confirmed by exploring the chemical space around them and revealing less active compounds bearing same structural moieties.

### Case study 2: NCI AIDS antiviral screen

The NCI AIDS Antiviral Screen data set [22] provides measures of the ability of chemical compounds to protect cells against the cytopathic effects of HIV (see Weislow et.al. [23]). The library consists of more than 40000 compounds as well as chemical structure descriptions and screening results.

**Figure 7:**
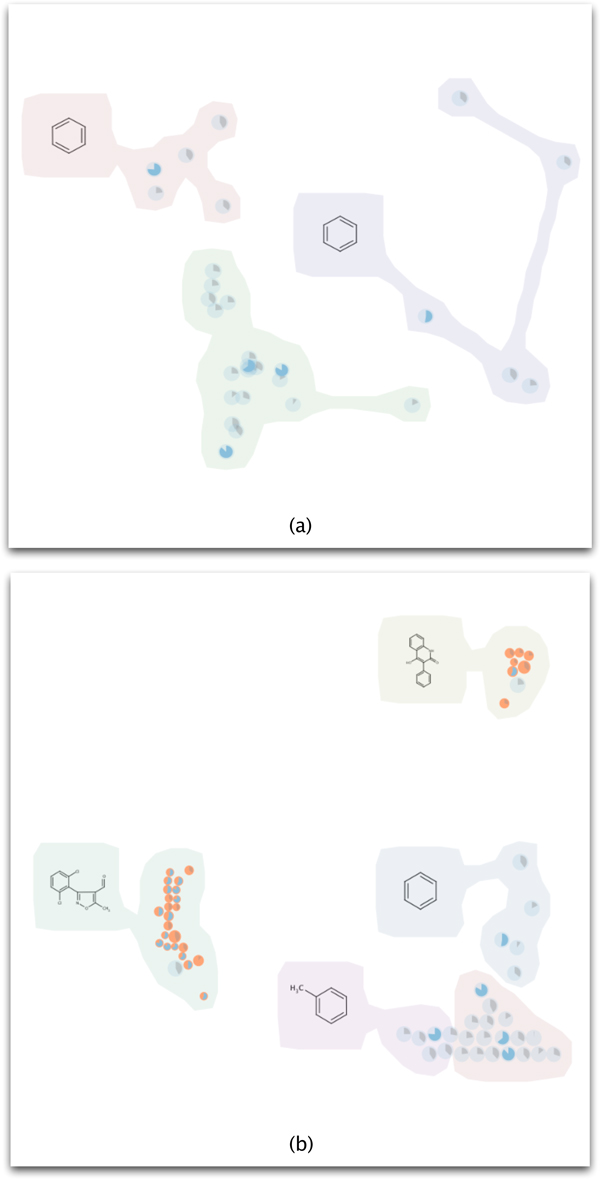
**Case study HIV**. Case Study HIV Antiviral Screen. Several compounds bearing a quinoline scaffold were identified with different activity level.

The 20 compounds that showed the highest activity level and high diversity were automatically preselected and projected in order to obtain an initial set of structures for hit selection. Within the projection view we get different clusters. By hovering over items we could see the chemical structure of each single compound. A cluster containing different heterocyclic molecules was selected and the detail view was used in order to find common substructures. The detailed view revealed a set of compounds containing benzothiazoles, quinazolines and quinolines, which are related structures. In order to confirm the selected compounds as active hits the project and expand option was used and the found cluster was investigated for common structures in the detailed view of HiTSEE. Several compounds bearing a quinoline scaffold were identified with different activity levels (Figure 7). Therefore we would suggest using this scaffold as a true hit and as a lead structure for further synthesis to establish SARs. Even though the confirmed active compounds of the published results of the screen did not list quinolines as active compounds, the related N-heterocyclic benzodiazepines, thiazolobenzimidazoles and pyrimidne analogues were confirmed active hits. Further details and the mode of action of these compounds can be found in De Clerq [24].

## Lessons learned

In this section we highlight two main lessons we have learned during the design of HiTSEE. The first lesson concerns the collaboration process between computer science experts and biochemistry experts, the second concerns the design strategy of HiTSEE.

During our collaboration we noticed that not only is it necessary to make sure that all the parties get acquainted with their respective research background and goals (e.g., the computer scientists have to understand the domain problem, the biochemists have to picture the capabilities that computer systems can offer) but also that their collaboration and influence rely on a steady exchange of ideas. In our experience it is not sufficient to meet at scheduled times in more or less formal meetings to report on the state of the work. A tighter integration is needed. Our project experienced big leaps every time we had computer scientists and biochemists sitting next to each other and walking through the data analysis steps together for extended periods of time.

From a visualization design point of view we learned that trying to achieve an overview of the whole data at all costs is not always the best strategy. Before developing HiTSEE we provided the users with a number of prototypes based on the idea of visualizing the entire chemical library under observation, or large portions of it. All our attempts in this direction failed because we misinterpreted the needs of our collaborators. The bottleneck in their analysis was not in spotting elements of interest as a way to kick-start the process, but rather to effectively and efficiently explore a fairly large number of compounds similar to a few selected ones.

We believe that visualization researchers and designers should take this advice into serious consideration and always ask if creating an overview is the best strategy to cope with the current problem. Especially when dealing with large data sets, trying to obtain full overviews might end up being not only impractical, but also not useful (or sub-optimal).

## Conclusion and future work

We presented HiTSEE (KNIME) a visualization tool for the analysis of high-throughput screening data for biochemistry experiments. HiTSEE proposes a smooth interface and interaction paradigm enabling the chemical space to be explored, find relationships between activity values and molecular structures. The paper presented a series of requirements, their impact on the design of the tool, and its effectiveness through a case study.

There are a number of issues we plan to address in the future. The projection view changes abruptly when modified by a number of external events, making it difficult to preserve the mental map of the projected items. We plan to develop methods to reduce the changes from one view to another and to implement smooth animations that help relate the new projection to the old one. As the analysis gets more complex and the user goes through multiple steps, it becomes difficult to remember previous steps and return to interesting states previously visited. We plan to implement a history and save mechanism that support this specific need. An assistance system to allow the user to choose a valuable expansion size is planned. An in-depth investigation of how different fingerprint generation methods and parameter sets influence the resulting distances is of high interest.

## Authors' contributions

HS, EB, JB, DM, TM wrote the text sections. HS and EB developed and programmed the prototype. HS integrated HiTSEE into KNIME. DM, TM, UG, OD revised the content critically and improved writing style.

## Competing interests

The authors declare that they have no competing interests.

## Additional information

The free version of HITSEE KNIME and an example configuration will be available at http://hitsee.hs8.de.
